# Supporting and Hindering Factors in the Use of Nursing Diagnosis in Romanian Healthcare: A Multilevel Regression Analysis

**DOI:** 10.1155/jonm/1543261

**Published:** 2026-08-12

**Authors:** Horațiu Rusu, Laura Elena Gligor, Carmen Daniela Domnariu, José Manuel Romero-Sánchez

**Affiliations:** ^1^ Centre for Social Research, Faculty of Social Sciences and Humanities, Lucian Blaga University of Sibiu, Sibiu, Romania, ulbsibiu.ro; ^2^ Social-Science Department, Faculty of Law and Social Science, “1 Decembrie 1918” University of Alba Iulia, Alba Iulia, Romania, uab.ro; ^3^ Dental Medicine and Nursing Department, Faculty of Medicine, Lucian Blaga University of Sibiu, Sibiu, Romania, ulbsibiu.ro; ^4^ Nursing and Physiotherapy Department, Faculty of Nursing and Physiotherapy, Instituto de Investigación e Innovación Biomédica de Cádiz (INiBICA), Universidad de Cádiz, Cádiz, Spain, uca.es

**Keywords:** behavior, nursing diagnosis, nursing education, nursing practice, self-directed learning

## Abstract

**Background:**

In an increasingly complex healthcare system, the use of nursing diagnosis is considered a gold standard of care and has become more integrated into clinical practice. Many countries have introduced policies to support the use of nursing diagnosis and align them with international nursing standards. In Romania, accreditation requirements have recently incorporated nursing diagnosis into clinical practice. However, although a legal framework exists, it remains unclear whether individual and organizational factors promote their use in daily practice.

**Aim:**

This study aims to examine the associations between individual factors (nurses’ education, attitudes, and self‐directed learning) and organizational factors (department size and type) and the use of nursing diagnosis in clinical practice, while accounting for theoretically relevant confounders.

**Methods:**

A cross‐sectional survey was conducted among clinical and managerial nurses in Romanian hospitals. A total of 691 nurses participated, and the final sample included 589 participants. Data were collected using a multistage sampling strategy and analyzed using multilevel regression modeling.

**Results:**

Multilevel regression analysis showed that positive attitudes and formal education were significantly associated with nursing diagnosis use, while lack of formal education or reliance on self‐training was associated with lower use. Greater time dedicated to self‐directed learning was associated with increased use. Older age, male gender, and larger department size were negatively associated with nursing diagnosis use, while use was lower in emergency and intensive care units compared to medical departments.

**Conclusions:**

Both individual and organizational factors are associated with the use of nursing diagnosis. Education, self‐directed learning, and positive attitudes support their use, while department characteristics, older age, and male gender are associated with lower use. Targeted training and the promotion of self‐directed learning may enhance the use in clinical practice.

**Implications for Nursing Leadership:**

Nurse leaders should promote nursing diagnosis‐focused education and continuing professional development tailored to nurses’ prior training, support opportunities for self‐directed learning, and implement structural measures in larger departments and adapted protocols in high‐acuity settings where lower use was observed.

## 1. Introduction

Documenting the nursing problems is crucial for demonstrating the contribution of nursing care to patient outcomes [1] and highlighting its role in the overall patient health [2]. According to the nursing process, which is the method for delivering nursing care in clinical practice, patient problems, risks, or strengths are identified in the second phase, referred to as nursing diagnosis (ND). ND encompasses a system of diagnostic categorization and describes the cognitive processes nurses use to make clinical judgments [3]. These clinical judgments made by nurses regarding the responses of individuals, families, or communities to health conditions or life processes, forming the basis for selecting nursing interventions within their scope of competence, are also named NDs [4].

The growing body of nursing research has demonstrated the impact of ND on two critical healthcare dimensions: patients and organizations. In terms of patient outcomes, studies have documented reductions in hospital mortality [5–7] and improvements in quality of life [8–10]. Additionally, clinical trials have shown that ND‐based nursing care plans can reduce anxiety and enhance anxiety self‐control in psychiatric patients [11] and alleviate cancer‐related fatigue in lung cancer patients [12]. The organizational impact of ND has been highlighted in two systematic reviews [13, 14], which emphasize that hospital readmission rates have decreased in medical, surgical, and emergency departments (ER) [6, 15]. Other studies have also demonstrated that implementing ND‐based care plans reduces the length of stay [2, 5, 16–20]. Furthermore, ND is a foundation for resource allocation and billing [21, 22]. ND documented in patient records facilitates more efficient hospital management, allowing for a more accurate assessment of nursing workload [23, 24] and, consequently, enabling the optimal allocation of nursing resources, including nurse staffing levels and skill mix [2].

Despite the internationally recognized benefits of ND, further investigation is needed into nurses’ behavior regarding ND, especially in healthcare settings where its use is in the early stages. In Romanian practice, for example, the nursing process began in 2018, and hospitals adopted it to meet hospital accreditation criteria [25]. Only two studies in Romania addressed nurses’ beliefs about ND in clinical practice [26–28]. Findings from these studies underscore the need for targeted educational programs and highlight the significant role of education in facilitating the use of ND. Additionally, the results indicate managerial support from head nurses and generally positive attitudes among nurses toward the use of ND in daily practice. However, while these studies provide valuable insights into Romanian nurses’ beliefs and profiles related to ND, no research has yet examined the impact of various factors on nurses’ behavior in using ND in clinical practice.

Given the importance and influence of using ND in clinical practice and the requirement for nurses to apply it, assessing the key aspects influencing their decisions to integrate ND into daily practice is essential. Gaining a deeper understanding of these elements will enable policymakers and hospital managers to design targeted interventions to enhance their use.

According to the theory of planned behavior (TPB), a widely used framework in health sciences for explaining and predicting healthcare provider behaviors [29], an individual’s intention to engage in a particular behavior is strongly influenced by their attitude toward it and by subjective norms—perceptions of social pressure to perform or avoid the behavior [30].

Numerous studies have shown that nurses’ attitudes toward ND play a significant role in its practical utilization. Moreover, these studies revealed that nurses with a positive attitude toward ND are more likely to use it compared to those with a negative or neutral attitude [31–34]. Therefore, attitudes have been recognized as a key factor in nurses’ utilization of ND [35]. Consequently, this study contends that positive nurses’ attitudes toward ND can potentially increase their use in clinical practice.

Continuing education has gained significant attention in nursing, particularly in light of the major changes over the last few decades [36]. One such change is the integration of standardized nursing languages into the nursing process, which differs from traditional nursing approaches [37]. Regarding ND specifically, its accuracy depends on intellectual and cognitive processes involving each nurse’s senses, intellect, and will [38]. As a result, ongoing and structured training programs have become essential for nurses to update their knowledge and maintain clinical competence [36]. The presence of ND programs has been identified as a key factor in the successful use of ND in clinical practice [1]. Several interventional studies have demonstrated a significant improvement in the accuracy of ND following focused educational training [39–41]. Targeted education on ND is likely to increase its utilization.

Self‐directed learning (SDL) is widely regarded as an effective approach for enabling practitioners to remain up to date with the current literature. In the context of continuous advances in medicine and biomedical sciences, healthcare professionals are required to develop the skills necessary for lifelong learning [42]. Moreover, SDL represents a key link between undergraduate education, postgraduate training, and continuing professional development, supporting the adoption of a reflective and critical approach to practice [43]. To incorporate new knowledge, a certain amount of time must be dedicated to learning activities. The effectiveness of learning is closely related to the time allocated to these activities, as evidence suggests that insufficient time has a direct negative impact on learning outcomes, affecting both knowledge acquisition and retention, and highlighting time as a key determinant of effective learning [44].

In nursing, attitude and the use of ND can be shaped by the characteristics of the nursing staff and healthcare institutions, which may either support or hinder their use [45].

Research suggests that smaller departments are generally more receptive to adopting ND [46]. However, nurses in ER often struggle to integrate ND into their daily practice due to the high patient volume and fast‐paced environment [47]. Similarly, another study examining nurses’ attitudes toward ND found low usage rates in ER settings [48]. Negative perceptions regarding the usefulness of ND have also been reported among intensive care unit (ICU) nurses [49]. As a result, this study suggests that the size and type of a department significantly influence ND utilization.

This paper identifies factors that could support or hinder the use of ND in clinical practice. The main objectives of this study were: (1) to examine whether specific education on ND and the time spent on SDL relates to nurses’ practical application of ND in clinical settings, (2) to examine the relationship between nurses’ attitudes toward ND, precisely their perceived usefulness and ease of use, and the practical application of ND in clinical settings, and (3) to assess if and how hospital department characteristics affect nurses’ behavior in using ND.

The following hypotheses were developed: (1) positive attitudes toward ND are significantly associated with its use in clinical practice; (2) targeted education is positively associated with their practical application; (3) greater time dedicated to SDL on ND is associated with increased use of ND; (4) the use of ND is negatively associated with hospital department size; (5) the use of ND is lower in ER and ICU compared to medical departments.

## 2. Materials and Methods

### 2.1. Design

A cross‐sectional survey design was used.

### 2.2. Population and Sampling

The participants were recruited between March to July 2022 and June 2024. The target population comprised all clinical and management nurses employed in Romanian hospitals. A multistage sampling strategy was employed. Ten hospitals from different counties were selected using a purposive sampling approach in the first stage. This selection ensured the representation of all the categories of hospitals, as classified by the Romanian Ministry of Health based on their level of competence and the complexity of medical services provided [50]. In the second stage, a convenience sampling approach was used, and the survey was distributed to all nurses working in each participating hospital by the nurse directors. Six hundred ninety‐one respondents from various departments across six hospitals (see Table A1 in Appendix A) accepted the invitation to complete the questionnaire. The department types from each hospital are listed in Table A2 in Appendix A. A nonprobabilistic convenience sample of 589 nurses was ultimately obtained after excluding cases with incomplete responses (*n* = 25) and observations from departments with fewer than five cases (*n* = 77), as detailed in the Data Collection section. The final sample comprised 589 nurses grouped into 42 departments across five hospitals. Hospital 6 was excluded from the analysis due to the small number of cases.

### 2.3. Data Collection

This study used an online platform to collect data. The invitation containing a link to the questionnaire, the study’s aims, a consent letter, the expected time required to complete the survey, and information on ethical considerations was distributed to all nurses via email or message application. Submission of a completed questionnaire was considered as consent to participate. Participants filled in the questionnaire anonymously.

### 2.4. Instrument and Measurement

The questionnaire was adapted from an instrument [51] developed to measure nurses’ beliefs about ND. The Italian version of the questionnaire was translated into Romanian, considering the linguistic similarities between the two languages. The translation process followed the methodology proposed by Beaton et al. [52]. Two independent Romanian translators produced forward translations: one with knowledge of the instrument’s concepts and one without. The two versions were then reconciled into a single Romanian version. This version was independently back‐translated into Italian by two professionals: a Romanian nurse with over 20 years of clinical experience in Italy and a certified Italian translator. The back‐translations were compared with the original version, and discrepancies were addressed through consensus within an expert committee, with adjustments made to ensure cultural relevance and clarity. A pretest was conducted using a test–retest approach with two groups of three experienced nurses. Based on their feedback, minor revisions were made to the wording of one item, and the time required for questionnaire completion was assessed [52]. The Romanian version of the instrument has been previously used in another study [26, 27], in which convergent validity was supported by statistically significant Spearman correlations among all belief scales (r ∼ s ∼ ranging from 0.442 to 0.596, *p* < 0.01), and internal consistency was confirmed with Cronbach’s alpha values of 0.96, 0.93, and 0.89 for the behavioral, normative, and control beliefs scales, respectively.

The items used in this study assess the following variables: nurses’ behavior regarding the practical application of ND in clinical settings (as an outcome variable), nurses’ attitudes toward ND, education provided on ND, and sociodemographic and contextual variables (as explanatory variables).

The practical application of ND was measured using an item that evaluated the frequency with which NDs are employed in clinical practice. Participants responded on a five‐point Likert scale, ranging from “1” (never) to “5” (always). The exact wording of the item was as follows: “How often do you use ND in your practice?”

Attitudes toward ND were measured using two items. The first item required nurses to evaluate the usefulness of ND in practice, while the second item assessed their evaluation of the ease of using ND in clinical settings. The exact wording of the questions is: “How useless/useful do you think the use of ND in clinical practice is? Please provide a score from ‘0’ to ‘10’, where ‘0’ represents ‘useless’ and ‘10’ represents ‘useful’”, and “Please give a grade of ‘0’ to ‘10’ for how difficult/easy it is for you to use the ND in clinical practice, where ‘0’ means ‘difficult’ and ‘10’ means ‘easy’”. The measurement scale employs a semantic differential approach to evaluate nurses’ attitudes toward ND. Each item presented two opposing descriptors (useless/useful, difficult/easy) representing contrasting views of ND activities. Responses were recorded on an 11‐point Osgood scale, with scores ranging from “0” (denoting a highly negative attitude) to “10” (representing a highly positive attitude). For analysis, the responses were recorded on a 5‐point scale, where “1” indicates a negative attitude and “5” a positive attitude. Specifically, responses of “0” or “1” were recoded as “1”; “2” or “3” as “2”; “4”, “5,” or “6” as “3”; “7” or “8” as “4”; and “9” or “10” as “5”.

ND education encompasses familiarity with ND concepts, formal training on ND, and self‐guided study. The scale comprises five categories: “1” education was focused on ND; “2” education also included ND; “3” education did not include ND; “4” participation at courses EMC; “5” self‐training on ND. Self‐training on ND captures nurses whose prior ND‐related education consisted exclusively of independent self‐study, with no formal undergraduate or continuing education on ND.

Sociodemographic and professional information includes data on participants’ age (years), gender (M/F), level of nursing education (measured on a two‐point scale—“1” high‐school/post‐secondary degree in nursing; “2” university degree in nursing), years of professional experience (self‐reported number of years of work experience as a nurse), current job role (measured on a three‐point scale: “1” junior and registered nurse; “2” senior registered nurse; and “3” lead nurse); participants workplace is a clinical hospital (“1” yes, “0” no). SDL on ND was defined as the number of hours per month that nurses devoted to individual learning activities related to ND outside formal education or organized training programs. Unlike self‐training on ND, which reflects prior educational background, this variable captures current learning behavior.

The contextual‐level variables included department size (i.e., the number of nurses working in the department) and categorization of departments based on Ministerial Order No. 1509/2008, which establishes the Nomenclature of Medical, Dental, and Pharmaceutical Specialties for the Healthcare Network [53]. Considering that, under Ministerial Order No. 446/2017, the ER and ICU departments were only recommended, rather than required, to use ND, a categorical variable was created, coded as follows: “0” for medical specialties, “1” for surgical specialties, and “2” for ER and ICU.

### 2.5. Method and Data

The method employed was multilevel linear regression, the maximum likelihood estimation method (the *xtmixed* routine). A multilevel design was appropriate since respondents were grouped into 78 departments within the six hospitals. Responses (i.e., 25 respondents) of nurses who did not report their departmental affiliation were excluded from the analysis. Given that the mixed‐effects regression literature recommends minimum thresholds for the number of groups and cases per group [54, 55], all observations from departments with fewer than five cases (i.e., 77 respondents from 34 departments) were further removed. The remaining sample comprises 589 nurses grouped into 42 departments across five hospitals. Since the number of hospitals was too low to be modeled separately, it was incorporated as an individual‐level variable to differentiate between participants’ workplaces. Therefore, the multilevel model employed in our analysis comprised the individual (nurse) and department levels. To account for the hierarchical structure of the data—nurses (Level 1) are nested within hospital departments (Level 2)—we employed multilevel linear modeling. This approach addresses the nonindependence of observations within the same unit, preventing the underestimation of standard errors [56, 57]. The null model indicated an intraclass correlation coefficient (ICC) of approximately 0.27, showing that a considerable proportion of the variance in ND behavior was attributable to differences between departments. The robustness of this approach was further confirmed through sensitivity analyses using different estimation methods (REML) and inclusion thresholds (see Appendix, Tables A3 and A4). Five multilevel regression models were constructed, progressively introducing the explanatory variables or groups of variables of interest. Statistical data analysis was performed with STATA 14. The results were considered significant if *p* was < 0.05.

### 2.6. Ethical Considerations

Ethical approval was obtained from each Research Ethics Committee of all the settings included in the study (approval no. 5998/9.03.2022; approval no. 2320/8.03.2022; approval no. 1580/22.03.2022; approval no. 14.705/22.03.2022; approval no. 16.758/10.03.2022) and Research Ethics Committee of the Institute for Quality of Life Research (ICCV), Romanian Academy (approval no. 77/09.02.2022). The respondents were informed about the study’s aim. Participants were guaranteed confidentiality and anonymity.

## 3. Results

### 3.1. Participants’ Characteristics

The sample included 589 valid observations. The main characteristics of nurses are described in Table 1. Department‐level characteristics are presented in Table 2. The sample comprised 589 (91%) female participants with a mean age of 43.36 (SD 8.11). Nurses had a mean of 16.22 years (SD 8.79) of professional experience. Regarding education in nursing, more than a quarter (27.16%) of participants hold a university degree. The lead nurses represent almost 8.32% of the sample. The proportion of nurses who benefited from education focused on ND was 13.92%, but 9.68% declared that they received no education on ND.

**TABLE 1:** Nurses’ sociodemographic and professional characteristics (*N* = 589).

Variables		Mean (SD)	Min./max.	*n* (%)
Gender	Female			536 (91.00)
	Male			53 (9.00)

Age		43.36 (8.11)	22/69	

Professional experience		16.22 (8.79)	< 1/> 25	

Education in nursing	HS/post‐SD degree			429 (72.84)
	University degree			160 (27.16)

Current job role	Junior and registered nurse			136 (23.09)
	Senior registered nurse			404 (68.59)
	Lead nurse			49 (8.32)

Education on ND	Education was focused on ND			82 (13.92)
	Education also included ND			299 (50.76)
	Education did not include ND			57 (9.68)
	Participated in courses EMC			84 (14.26)
	Self‐training on ND			67 (11.38)

Self‐directed learning on ND	Number of hours per month	2.51 (3.66)	0/> 10	

Works in a clinical hospital	Yes			395 (67.06)
	No			194 (32.94)

*Note:* HS/post‐SD degree = high school/post‐secondary degree.

**TABLE 2:** Department‐level characteristics.

Department characteristics		Mean (SD)	Min./max.	*n* (%)
Specialty type	Medical			267 (45.33)
	Surgical			286 (48.56)
	ER and ICU			36 (6.11)

Number of nurses per department		32.78 (29.67)	5/85	

### 3.2. Behavior and Attitudes

First, a descriptive analysis was performed on the dependent variable. The findings revealed that 57.05% participants use ND always or often, whereas 27.84% stated they rarely or never used it, and 15.11% reported occasional use (*M* = 3.41, SD = 1.35).

Regarding the usefulness of ND, the data revealed that 61.63% nurses view ND use as useful, while 18.68% considered it not useful, and 19.69% remained neutral. The overall rating yielded a mean of 3.7 (SD = 1.35). Concerning the ease of use of ND, data indicate that 52.12% respondents find ND use relatively straightforward, 22.58% consider it challenging, and 25.30% remain neutral. The overall rating yielded a mean of 3.43 (SD = 1.29).

### 3.3. Multilevel Regression Models Predicting Nurses’ Behavior Toward ND

Table 3 presents the findings of the multilevel regression. The null model (Model 0) delineates the variance distribution across levels. The overall mean level of ND use across the 42 departments was 3.49 (SD = 0.12). In this model, only random effects for the intercept at level 2 were included (SD = 0.73), indicating significant variability in department‐specific intercepts. ICC was calculated at 0.265, suggesting that 26.5% of the total variability in ND use is attributable to differences between departments.

**TABLE 3:** Multilevel regression model of ND behavior.

	Null model	Model 1	Model 2	Model 3	Model 4	Model 5
		Level 1 variables only			Level 1 and 2 variables	
	B/(SE)	B/(SE)	B/(SE)	B/(SE)	B/(SE)	B/(SE)
Intercept	3.491^∗∗∗^ (0.129)	2.293^∗∗∗^ (0.187)	2.646^∗∗∗^ (0.238)	3.479^∗∗∗^ (0.382)	4.205^∗∗∗^ (0.464)	4.190^∗∗∗^ (0.462)
Usefulness		0.112^∗^ (0.056)	0.092^a^ (0.055)	0.107^∗^ (0.054)	0.112^∗^ (0.054)	0.118^∗^ (0.054)
Ease of use		0.226^∗∗∗^ (0.058)	0.185^∗∗^ (0.057)	0.186^∗∗∗^ (0.056)	0.184^∗∗∗^ (0.056)	0.183^∗∗∗^ (0.056)
Education was focused on ND (ref.)						
Education also included ND			−0.135 (0.146)	−0.188 (0.143)	−0.207 (0.142)	−0.208 (0.142)
Education did not include ND			−1.030^∗∗∗^ (0.209)	−1.012^∗∗∗^ (0.207)	−1.028^∗∗∗^ (0.206)	−1.013^∗∗∗^ (0.206)
Participated in courses EMC			−0.227 (0.186)	−0.227 (0.183)	−0.231 (0.182)	−0.220 (0.182)
Self‐training on ND			−0.540^∗∗^ (0.198)	−0.524^∗∗^ (0.195)	−0.539^∗∗^ (0.194)	−0.522^∗∗^ (0.194)
No. of hours/month of self‐directed learning on ND			0.046^∗∗∗^ (0.014)	0.046^∗∗∗^ (0.013)	0.048^∗∗∗^ (0.013)	0.048^∗∗∗^ (0.013)
University degree in nursing			0.049 (0.112)	0.060 (0.112)	0.058 (0.112)	0.054 (0.112)
Professional experience				0.002 (0.009)	0.002 (0.009)	0.002 (0.009)
Junior and registered nurse (ref.)						
Senior registered nurse				0.067 (0.143)	0.066 (0.143)	0.075 (0.143)
Lead nurse				0.007 (0.232)	0.002 (0.231)	−0.006 (0.231)
Age				−0.017^∗^ (0.009)	−0.017^∗^ (0.009)	−0.018^∗^ (0.009)
Male				−0.848^∗∗∗^ (0.166)	−0.839^∗∗∗^ (0.166)	−0.853^∗∗∗^ (0.166)
Works in a clinical hospital				−0.268 (0.211)	−0.108 (0.204)	−0.160 (0.198)
Department size					−0.043^∗^ (0.016)	−0.033^∗^ (0.016)
Medical specialty (ref.)						
Surgical specialty						−0.165 (0.197)
ER and ICU						−0.652^a^ (0.353)
Random part						
SD (intercept department)	0.731^∗∗∗^ (0.113)	0.627^∗∗∗^ (0.101)	0.591^∗∗∗^ (0.096)	0.568^∗∗∗^ (0.096)	0.502^∗∗∗^ (0.091)	0.473^∗∗∗^ (0.089)
Level 2 variance	0.534	0.393	0.349	0.323	0.252	0.223
Level 1 variance	1.478	1.345	1.254	1.196	1.196	1.196
ICC	26.56%	22.60%	21.75%	21.25%	17.42%	15.78%
N	589	589	589	589	589	589
ll (Log‐likelihood)	−984.002	−952.733	−931.676	−917.111	−913.664	−911.974
Deviance	1968.004	1905.466	1863.352	1834.222	1827.328	1823.948
BIC	1987.139	1937.358	1933.514	1942.656	1942.140	1951.517
AIC	1974.004	1915.466	1885.351	1868.223	1863.328	1863.949

*Note:* “ref.” = reference category.

^∗^ *p* < .05.

^∗∗^ *p* < .01.

^∗∗∗^ *p* < .001.

^a^ *p* < .10.

In Models 1–3, the individual‐level variables’ relationship to nurses’ practical application of ND in clinical settings was gradually examined, thereby testing our first (H1), second (H2), and third (H3) hypotheses.

Model 1 showed a significant positive effect of attitudes toward ND on its practical application in clinical settings, with *b* = 0.112 for usefulness and *b* = 0.226 for ease of use, thereby validating our first hypothesis. Compared to the null model, the individual‐level variance decreased to 1.345, while the department‐level variance reduced to 0.393.

Model 2 tested the second (H2) and third (H3) hypotheses by incorporating the additional effects of dedicated ND education and training. The results show that education plays a critical role in ND use. Nurses whose formal education did not include ND (*b* = −1.030) and those who relied on self‐training (*b* = −0.540) exhibited a significantly lower likelihood of using ND in practice compared to those who received formal education focused on ND, thereby validating the second hypothesis (H2). Notably, ND use significantly increased with the number of hours of SDL on ND (*b* = 0.046). At the same time, the level of educational degree did not have a significant impact, thus validating the third hypothesis (H3). Attitudes toward ND continued to exert a significant positive effect in Model 2. Compared to Model 1, the individual‐level variance decreased to 1.254, and the department‐level variance diminished to 0.349.

Model 3, which controlled for sociodemographic and professional variables, indicated that both attitudes and education maintained their significant effects on ND use. Additionally, this model revealed a significant negative effect of age (*b* = −0.017) and a significantly lower likelihood of ND use among men (*b* = −0.848) than women.

Compared to Model 2, the individual‐level variance decreased to 1.196 and the department‐level variance diminished to 0.323.

Models 4 and 5 introduced department‐level variables. First, the effect of department size is tested, followed by the effect of department type, thereby verifying the final two hypotheses (H4 and H5). In both Models 4 and 5, the significant effects indicated in the previous models are maintained. Model 4 reveals a significant negative relationship between the number of nurses in a department and ND practical application, with a coefficient of −0.043. In other words, as the number of nurses in a department increased, the use of ND in practice decreased, thereby supporting H4. Compared to the previous model, the department‐level variance diminished to 0.252. The last model reveals the effect of the category of department. ER and ICU departments were less likely to use ND in practice than medical departments (*p* = 0.1), providing marginal evidence supporting H5. Compared to Model 4, the department‐level variance diminished to 0.223.

## 4. Discussion

The findings of this study highlight that positive attitudes toward ND, along with focused education on ND, are key factors in the use of ND in clinical practice. Additionally, SDL on ND also plays a significant role in the use of ND. The findings of our study are consistent with prior research showing a strong direct relationship between attitudes and behavior [31, 33, 51]. The critical role of ND education was also emphasized in other studies, which highlight that without proper nurse training and the development of critical thinking skills, the quality of the nursing process remains low [41, 58]. In addition, evidence shows that continuing education interventions can significantly improve nurses’ critical thinking, clinical reasoning, and diagnostic accuracy, thereby enhancing the use of ND in practice [59]. Recent evidence shows that educational and technological interventions, such as clinical decision support systems, can significantly improve ND accuracy and support the development of clinical reasoning skills, which are essential for the effective use of ND [60]. Interventional studies conducted on samples from Brazil [61] and Switzerland [62] involving nurses with different levels of ND education demonstrated that educational intervention programs significantly improved the use and quality of the nursing process, including ND. Furthermore, SDL has been shown to improve knowledge acquisition, clinical skills, and professional competencies among nurses, supporting their overall development and contributing to the formation of professional identity [63–65]. Our results highlight, in particular, a significant association between SDL and the use of ND, suggesting its important role in the long‐term professional preparation and continuous development of nurses.

Additionally, our analysis provides deeper insights into the characteristics of the nursing staff and healthcare institutions. The results of the present study show a significant relationship between the number of nurses in a department and the practical application of ND. A higher number of nurses in the department was associated with a lower frequency of ND use. One possible explanation for the reduced use of ND is the complexity involved in their formulation, as well as nurses’ lack of familiarity with their structure. For example, nurses may be unfamiliar with identifying related factors, and the process is often perceived as time‐consuming [66]. Furthermore, when analyzing the Romanian context, evidence suggests that despite the large amount of data collected by nurses in nursing documentation, these data were rarely used to formulate NDs [26, 27]. Additionally, data collection was based on a single nursing model (the Henderson model) across hospital settings, as recommended by the National Authority of Quality Management in Health (NAQMH) [25] in alignment with the Romanian nursing curriculum [67]. In this context, it is possible that nurses had limited experience in applying clinical reasoning to formulate NDs, or that the data collected were not sufficiently relevant for identifying appropriate diagnoses. However, beyond individual factors, implementation‐related and organizational factors also play a significant role. Previous research indicates that smaller departments are more likely to use ND than larger ones, likely due to structural characteristics. Greater fragmentation of care in larger units may hinder ND use, whereas smaller units allow for better continuity of care and greater control over clinical practice [46].

Significant differences in the use of ND were observed among hospital departments.

Lower use of ND was observed in emergency room (ER) and ICU departments compared to medical departments, though this difference reached only marginal statistical significance (*p* < 0.10). These findings are consistent with previous studies highlighting difficulties in integrating ND into high‐paced ER environments [47, 68], although some evidence shows that ND can be effectively used in ER settings and improve patient care quality [69]. Regarding ICUs, findings in the literature are inconsistent: some studies report higher ND use and associations with patient outcomes such as length of stay and mortality [5, 48, 70], while others highlight negative perceptions among nurses [49]. Explanations based solely on workload and time constraints are not supported by all evidence, as ND is also used in other time‐constrained settings [71, 72]. A possible explanation for these discrepancies may be related to institutional policies.

In analyzing the relationship between nurses’ demographic and professional variables and the utilization of ND, age and gender emerged as the only factors with significant differences. Regarding age, our findings suggest a negative association between age and ND utilization, meaning that older nurses were less likely to use ND. These results align with those of D’Agostino et al. [51], who conducted a comparative study in Italy and Spain and reported similar trends. However, our findings contradict those of Lumillo‐Gutiérrez et al. [45], who observed that older nurses had higher ND utilization rates. These authors attributed their results to financial incentives for permanent staff to comply with care plans in various Spanish healthcare services. Given that job stability in Spain often occurs later in a nurse’s career, experienced nurses may be more motivated to use ND due to these incentives. In contrast, in Romania, where such incentive systems are not in place, a reasonable explanation for our findings is the recent introduction of ND into clinical practice. Although ND has been part of Romanian nursing education for over 30 years, many older nurses appear unfamiliar or uncomfortable with its application in daily practice.

Regarding gender differences, our study found that male nurses were less likely to use ND than female nurses. However, this finding contrasts with Kamberi [48], who reported that female nurses perceived greater difficulties in applying ND in clinical practice than their male counterparts. These discrepancies suggest that gender‐related factors influencing ND utilization may be context‐dependent and shaped by role expectations according to the workplace culture. The distribution of tasks within nursing may imply that the observed differences in ND utilization are not directly attributable to gender itself but rather to the type of responsibilities assigned to nurses based on their gender.

### 4.1. Strengths, Limitations, and Future Research

This study has several strengths and limitations that should be considered when interpreting the findings.

To minimize potential self‐report bias, the questionnaire was administered online with fully anonymous responses and focused on concrete behaviors. The instrument underwent a cross‐cultural adaptation process. While some variables were measured using single items, the presence of department‐level variation and the consistent associations with theoretically relevant factors—such as education focused on ND, self‐training, SDL, and perceived ease of use—support the validity of the measure.

However, convenience sampling limits the generalization of this study’s results. Self‐assessment instead of objective measurements should be considered, particularly when evaluating the actual use of ND in clinical practice. The fact that the nurse director was responsible for recruitment may have influenced nurses’ willingness to complete the questionnaire.

Finally, the model considered only a limited number of factors related to the use of ND. As stated, other factors, such as workload level, the volume of administrative tasks assigned to nurses, type of management, types of specialties within the hospital, staff‐to‐patient ratios, and level or type of funding, could not be addressed due to a lack of information. Although we did not have sufficient data to measure this with certainty, it is possible that hospital‐level factors also generate some variability. Future research, considering the influence of additional factors on the use of ND, should be explored with regression analyses.

## 5. Implications for Nursing Leadership

The findings of this study provide several implications for nursing leadership and management aimed at promoting the use of ND in clinical practice.

Given the positive association between formal education, SDL, and ND use, nurse managers should support structured educational programs, continuing professional development, and protected learning opportunities for nursing staff. Educational initiatives should be tailored according to nurses’ previous exposure to ND, with particular attention given to nurses educated before the integration of ND into nursing curricula. Beyond knowledge acquisition, leadership strategies should explicitly address the attitudinal dimension of ND, including its perceived usefulness and ease of use, through peer‐learning activities, mentorship, and case‐based educational approaches.

Nursing leaders should also foster a supportive practice environment that values critical thinking, clinical reasoning, and the use of standardized nursing languages. Creating a positive organizational culture that values ND, allocating time for learning activities, and ensuring access to educational resources may enhance the integration of ND into clinical practice and support the delivery of high‐quality nursing care.

Given the lower use of ND observed in larger departments, nursing leaders may consider structural solutions, such as appointing designated ND coordinators or nurses with expertise in ND, to support implementation and reduce the effects of care fragmentation. Similarly, the lower use identified in ER and ICU suggests the need for adapted protocols and implementation strategies that address the specific workflow and clinical demands of these settings. The associations identified with age and gender indicate that workforce characteristics should also be considered when designing implementation and educational interventions.

Finally, regular departmental audits of ND documentation and use could provide valuable feedback to nurses and managers, identify areas requiring improvement, and monitor progress over time. Audit findings may be used to guide targeted training initiatives and quality improvement strategies. By combining education, organizational support, and systematic monitoring, nursing leaders can enhance the implementation of ND and contribute to higher‐quality, evidence‐based nursing care.

## 6. Conclusion

This study provides evidence regarding the factors that influence the use of ND in clinical practice. While specific education, the amount of time dedicated to SDL, and nurses’ attitudes enhance the use of ND, department size and type are factors that affect their utilization. Additionally, older age and male gender were associated with a lower likelihood of ND use. By identifying both individual and organizational predictors within a multilevel framework, this study contributes to the evidence base needed to design effective implementation strategies in healthcare systems at similar stages of nursing practice development.

## Author Contributions

Horatiu Rusu: conceptualization, methodology, formal analysis, investigation, validation, data curation, writing–original draft preparation, writing–review and editing, funding acquisition. Laura Elena Gligor: conceptualization, methodology, formal analysis, investigation, validation, data curation, writing–original draft preparation, writing–review and editing. Carmen Daniela Domnariu: conceptualization, methodology, formal analysis, investigation, validation, data curation, writing–original draft preparation, writing–review and editing. José Manuel Romero‐Sánchez: conceptualization, methodology, formal analysis, investigation, validation, data curation, writing–original draft preparation, writing–review and editing, supervision.

## Funding

This publication received financial support under Order No. 3876 of 5 May 2026 issued by the Romanian Ministry of Education.

## Disclosure

All authors read and approved the final manuscript.

## Conflicts of Interest

The authors declare no conflicts of interest.

## Supporting Information

Additional supporting information can be found online in the Supporting Information section.

## Supporting information


**Supporting Information 1** Appendix A. This appendix provides additional methodological information, including the classification of participating hospitals, the distribution of departments and respondents across hospitals, and sensitivity analyses conducted using alternative multilevel model specifications. Specifically, it includes hospital characteristics (Table A1), the distribution of departments included in the analysis (Table A2), and two supplementary multilevel regression models based on alternative department inclusion criteria (Tables A3 and A4).

**Supporting Information 2** Supporting File. Comparison of OLS and MLM on Identical Estimation Sample. This Supporting file presents a comparison between ordinary least squares (OLS) regression and multilevel modeling (MLM) using the same estimation sample (*N* = 589). The analyses were conducted to assess the robustness of the findings and to evaluate the impact of accounting for department‐level clustering on parameter estimates and model fit.

## Data Availability

The datasets generated and analyzed during the current study are available from the corresponding author upon reasonable request.
