# Supplementary material for: Supporting and Hindering Factors in the Use of Nursing Diagnosis in Romanian Healthcare: A Multilevel Regression Analysis
Source: J Nurs Manag. 2026 Aug 12;2026:1543261. doi: 10.1155/jonm/1543261 (PMC13469325; doi:10.1155/jonm/1543261)
Supplement: Supplementary file 1 [file JONM-2026-1543261-s001.docx]

**Appendix A**

**Table A1. Hospitals classification by the Romanian Ministry of Health (N = 691 initial sample)**

|  | Include in the analysis | | | | | Not included in the analysis |
| --- | --- | --- | --- | --- | --- | --- |
| Hospital | H1 | H2 | H3 | H4 | H5 | H6 |
| County | IS | PH | IS | SB | AB | BV |
| Ministry of Health  classification based on level of competence and the complexity of medical services | IV | III | II | II | III | V |
| Number of beds | 385 | 1160 | 1128 | 1054 | 763 | 121 |
| Clinical hospital | No | No | Yes | Yes | No | No |
| Nr. of respondents | 110 | 102 | 350 | 92 | 27 | 10 |

**Table A2. List of departments and number of answers per department in each of the five hospitals included in the analysis**

| Hospital  Department | H1 | H2 | H3 | H4 | H5 | Total |
| --- | --- | --- | --- | --- | --- | --- |
|  |  |  |  |  |  |  |
| Palliative care | 15 |  |  |  |  | 15 |
| Neurology |  |  |  | 7 |  | 7 |
| Infectious diseases | 8 | 5 |  |  |  | 13 |
| Internal medicine | 25 | 7 | 71 | 22 |  | 125 |
| Gastroenterology | 6 |  | 21 |  |  | 27 |
| ICU | 13 |  | 10 |  | 5 | 28 |
| Medical rehabilitation |  |  |  | 6 |  | 6 |
| General Surgery | 9 | 13 | 85 |  | 6 | 113 |
| Neonatology | 8 |  |  |  |  | 8 |
| Obstetrics gynecology | 5 | 5 |  | 9 |  | 19 |
| Vascular Surgery |  | 10 | 9 |  |  | 19 |
| Otorhinolaryngology |  | 14 | 16 |  |  | 30 |
| Plastic surgery |  | 5 | 47 |  |  | 52 |
| Neurosurgery |  | 10 |  | 6 |  | 16 |
| Oral and maxillofacial surgery |  |  | 9 |  |  | 9 |
| Nephrology |  | 6 |  |  |  | 6 |
| Ophthalmology |  | 9 |  |  |  | 9 |
| Orthopedics |  |  | 19 |  |  | 19 |
| Dermatovenerology |  | 5 |  |  |  | 5 |
| Hematology |  | 5 | 8 |  |  | 13 |
| Cardiology |  |  | 14 | 10 |  | 24 |
| Diabetology |  |  | 13 |  |  | 13 |
| Endocrinology |  |  | 5 |  |  | 5 |
| ER (Emergency Room) |  |  |  | 8 |  | 8 |
| TOTAL |  |  |  |  |  | 589 |

Note: each cell containing values represents one department included in the	analysis.

**Sensitivity analyses**

| Table A3. Multilevel regression model of ND behavior (REML) (> 5 cases per department; 42 departments; 5 hospitals) | | | | | | |
| --- | --- | --- | --- | --- | --- | --- |
|  | Null model | Model 1 | Model 2 | Model 3 | Model 4 | Model 5 |
|  |  | *Level 1 variables only* | | | *Level 1 & 2 variables* | |
|  | B /(SE) | B /(SE) | B /(SE) | B /(SE) | B /(SE) | B /(SE) |
| Intercept | 3.490*** (0.131) | 2.295*** (0.188) | 2.647*** (0.240) | 3.481*** (0.388) | 4.212*** (0.477) | 4.198*** (0.482) |
| Usefulness |  | 0.111* (0.056) | 0.092^+^ (0.055) | 0.107*  (0.055) | 0.112* (0.054) | 0.117* (0.055) |
| Ease of use |  | 0.226*** (0.058) | 0.184** (0.057) | 0.185*** (0.056) | 0.184*** (0.056) | 0.181*** (0.056) |
| Education was focused on ND (ref.) |  |  |  |  |  |  |
| Education also included ND. |  |  | -0.134 (0.147) | -0.187 (0.144) | -0.204 (0.144) | -0.203 (0.144) |
| Education did not include ND. |  |  | -1.028*** (0.210) | -1.009*** (0.209) | -1.022*** (0.209) | -1.003*** (0.209) |
| Participated in courses EMC |  |  | -0.227  (0.187) | -0.226  (0.185) | -0.230  (0.184) | -0.219  (0.184) |
| Self-training on ND |  |  | -0.540**  (0.199) | -0.524**  (0.197) | -0.538**  (0.197) | -0.522**  (0.197) |
| ND study hours/ month |  |  | 0.046*** (0.014) | 0.046*** (0.013) | 0.048*** (0.013) | 0.047*** (0.013) |
| University degree in nursing |  |  | 0.050 (0.113) | 0.060 (0.114) | 0.059 (0.113) | 0.055 (0.113) |
| Professional experience |  |  |  | 0.002 (0.009) | 0.002 (0.009) | 0.002 (0.009) |
| Junior & registered nurse (ref.) |  |  |  |  |  |  |
| Senior registered nurse |  |  |  | 0.067 (0.145) | 0.066 (0.144) | 0.075 (0.144) |
| Lead nurse |  |  |  | 0.010 (0.235) | 0.007 (0.234) | 0.004 (0.234) |
| Age |  |  |  | -0.017* (0.009) | -0.017* (0.009) | -0.018* (0.009) |
| Male |  |  |  | -0.848*** (0.168) | -0.839*** (0.168) | -0.851*** (0.168) |
| Works in a clinical hospital |  |  |  | -0.266 (0.218) | -0.105 (0.214) | -0.153 (0.1214) |
| Department size |  |  |  |  | -0.044** (0.017) | -0.033^+^ (0.018) |
| Medical specialty (ref.) |  |  |  |  |  |  |
| Surgical specialty |  |  |  |  |  | -0.170 (0.214) |
| ER & ICU |  |  |  |  |  | -0.660^+^  (0.382) |
| Random part |  |  |  |  |  |  |
| SD (Intercept department) | 0.745*** (0.115) | 0.639*** (0.103) | 0.603*** (0.099) | 0.592*** (0.100) | 0.538*** (0.097) | 0.532*** (0.099) |
| Level 2 variance | 0.555 | 0.409 | 0.364 | 0.351 | 0.289 | 0.283 |
| Level 1 variance | 1.478 | 1.349 | 1.272 | 1.224 | 1.223 | 1.222 |
| ICC | 27.29% | 23.24% | 22.24% | 22.28% | 19.11% | 18.82% |
|  |  |  |  |  |  |  |
| N | 589 | 589 | 589 | 589 | 589 | 589 |
| ll (Log-likelihood) | -985.125 | -958.261 | -945.763 | -942.846 | -942.781 | -941.982 |
| Deviance | 1970.25 | 1916.522 | 1891.526 | 1885.692 | 1885.562 | 1883.964 |
| BIC | 1989.385 | 1948.414 | 1961.689 | 1994.125 | 2000.374 | 2011.533 |
| AIC | 1976.250 | 1926.522 | 1913.526 | 1919.692 | 1921.562 | 1923.964 |
| *Notes: *p < .05; **p < .01; ***p < .001; +p < .10.; „ref.” = reference category* | | | | | | |

| Table A4. Multilevel regression model of ND behavior, (ML), (> 7 cases per department; 29 departments; 4 hospitals) | | | | | | |
| --- | --- | --- | --- | --- | --- | --- |
|  | Null model | Model 1 | Model 2 | Model 3 | Model 4 | Model 5 |
|  |  | *Level 1 variables only* | | | *Level 1 & 2 variables* | |
|  | B /(SE) | B /(SE) | B /(SE) | B /(SE) | B /(SE) | B /(SE) |
| Intercept | 3.543*** (0.120) | 2.477*** (0.196) | 2.757*** (0.255) | 3.882*** (0.412) | 4.518*** (0.490) | 4.505*** (0.489) |
| Usefulness |  | 0.099 (0.060) | 0.080 (0.059) | 0.102^+^  (0.058) | 0.101^+^  (0.058) | 0.104^+^  (0.058) |
| Ease of use |  | 0.202** (0.063) | 0.173** (0.061) | 0.166** (0.060) | 0.166** (0.060) | 0.166** (0.060) |
| Education was focused on ND (ref.) |  |  |  |  |  |  |
| Education also included ND. |  |  | -0.137 (0.158) | -0.193 (0.155) | -0.208 (0.154) | -0.213 (0.154) |
| Education did not include ND. |  |  | -0.933*** (0.226) | -0.864*** (0.223) | -0.885*** (0.222) | -0.882*** (0.222) |
| Participated in courses EMC |  |  | -0.167  (0.199) | -0.177  (0.194) | -0.182  (0.193) | -0.174  (0.193) |
| Self-training on ND |  |  | -0.549**  (0.209) | -0.526*  (0.205) | -0.543**  (0.205) | -0.534**  (0.205) |
| ND study hours/ month |  |  | 0.044** (0.015) | 0.046** (0.014) | 0.047*** (0.014) | 0.046** (0.014) |
| University degree in nursing |  |  | 0.061 (0.119) | 0.097 (0.119) | 0.092 (0.119) | 0.093 (0.119) |
| Professional experience |  |  |  | -0.001 (0.010) | -0.001 (0.009) | -0.001 (0.009) |
| Junior & registered nurse (ref.) |  |  |  |  |  |  |
| Senior registered nurse |  |  |  | 0.072 (0.151) | 0.069 (0.150) | 0.072 (0.150) |
| Lead nurse |  |  |  | -0.050 (0.251) | -0.057 (0.251) | -0.068 (0.251) |
| Age |  |  |  | -0.019* (0.009) | -0.018* (0.009) | -0.019* (0.009) |
| Male |  |  |  | -0.881*** (0.176) | -0.871*** (0.176) | -0.879*** (0.176) |
| Works in a clinical hospital |  |  |  | -0.556** (0.216) | -0.383^+^ (0.212) | -0.407^+^ (0.208) |
| Department size |  |  |  |  | -0.038* (0.016) | -0.033* (0.016) |
| Medical specialty (ref.) |  |  |  |  |  |  |
| Surgical specialty |  |  |  |  |  | -0.039 (0.199) |
| ER & ICU |  |  |  |  |  | -0.419  (0.345) |
| Random part |  |  |  |  |  |  |
| SD (Intercept department) | 0.543*** (0.106) | 0.519*** (0.101) | 0.524*** (0.102) | 0.459*** (0.099) | 0.403*** (0.094) | 0.384*** (0.094) |
| Level 2 variance | 0.294 | 0.269 | 0.275 | 0.211 | 0.163 | 0.148 |
| Level 1 variance | 1.515 | 1.392 | 1.306 | 1.236 | 1.235 | 1.236 |
| ICC | 16.27% | 16.21% | 17.38% | 14.58% | 11.63% | 10.67% |
|  |  |  |  |  |  |  |
| N | 519 | 519 | 519 | 519 | 519 | 519 |
| ll (Log-likelihood) | -863.549 | -841.553 | -825.841 | -809.346 | -806.782 | -806.043 |
| Deviance | 1727.098 | 1683.106 | 1651.682 | 1618.692 | 1613.564 | 1612.086 |
| BIC | 1745.854 | 1714.366 | 1720.453 | 1724.974 | 1726.098 | 1737.124 |
| AIC | 1733.099 | 1693.106 | 1673.682 | 1652.692 | 1649.564 | 1652.086 |
| *Notes: *p < .05; **p < .01; ***p < .001; +p < .10.; „ref.” = reference category* | | | | | | |
