# Supplementary material for: Supporting and Hindering Factors in the Use of Nursing Diagnosis in Romanian Healthcare: A Multilevel Regression Analysis
Source: J Nurs Manag. 2026 Aug 12;2026:1543261. doi: 10.1155/jonm/1543261 (PMC13469325; doi:10.1155/jonm/1543261)
Supplement: Supplementary file 2 [file JONM-2026-1543261-s002.docx]

**Supplementary file**

Comparison of OLS and MLM on identical estimation sample (N=589)

|  | (1) | (2) |
| --- | --- | --- |
|  | ND behavior | ND behavior |
|  | b/se | b/se |
| main |  |  |
| usefulness | 0.126^*^ | 0.118^*^ |
|  | (0.056) | (0.054) |
| ease of use | 0.201^***^ | 0.183^***^ |
|  | (0.058) | (0.056) |
| education was focused on ND | 0.000 | 0.000 |
|  | (.) | (.) |
| education included also ND | -0.255^+^ | -0.208 |
|  | (0.149) | (0.142) |
| education not included ND | -1.127^***^ | -1.013^***^ |
|  | (0.214) | (0.206) |
| participated at courses EMC about ND | -0.228 | -0.220 |
|  | (0.186) | (0.182) |
| self-training on ND | -0.532^**^ | -0.522^**^ |
|  | (0.203) | (0.194) |
| no. of hours/month of self-directed learning on ND | 0.053^***^ | 0.048^***^ |
|  | (0.014) | (0.013) |
| highschool & post-secondary highschool | 0.000 | 0.000 |
|  | (.) | (.) |
| university degree | 0.055 | 0.054 |
|  | (0.117) | (0.112) |
| professional experience | 0.005 | 0.002 |
|  | (0.009) | (0.009) |
| junior & registered nurse | 0.000 | 0.000 |
|  | (.) | (.) |
| senior registered nurse | 0.071 | 0.075 |
|  | (0.151) | (0.143) |
| lead nurse | -0.127 | -0.006 |
|  | (0.243) | (0.231) |
| age | -0.018^*^ | -0.018^*^ |
|  | (0.009) | (0.009) |
| female | 0.000 | 0.000 |
|  | (.) | (.) |
| male | -0.875^***^ | -0.853^***^ |
|  | (0.175) | (0.166) |
| works in a clinical hospital | -0.256^*^ | -0.160 |
|  | (0.126) | (0.198) |
| department size | -0.030^**^ | -0.033^*^ |
|  | (0.010) | (0.016) |
| medical specialty | 0.000 | 0.000 |
|  | (.) | (.) |
| surgical specialty | -0.105 | -0.165 |
|  | (0.105) | (0.197) |
| ER & ICU | -0.546^*^ | -0.652^+^ |
|  | (0.219) | (0.353) |
| Constant | 4.070^***^ | 4.190^***^ |
|  | (0.413) | (0.462) |
| lns1_1_1 |  |  |
| Constant |  | -0.748^***^ |
|  |  | (0.189) |
| lnsig_e |  |  |
| Constant |  | 0.090^**^ |
|  |  | (0.030) |
| N | 589.000 | 589.000 |
| Log-likelihood | -922.652 | -911.974 |
| AIC | 1881.304 | 1863.949 |
| BIC | 1960.116 | 1951.517 |

Note: MLM accounts for department-level clustering (LR test vs. linear model, p < .001).
